# Salpingitis Impairs Bovine Tubal Function and Sperm-Oviduct Interaction

**DOI:** 10.1038/s41598-019-47431-x

**Published:** 2019-07-26

**Authors:** Loveth E. Owhor, Sven Reese, Sabine Kölle

**Affiliations:** 1 School of Medicine, Health Sciences Centre, University College Dublin (UCD), Dublin, Ireland; 2 School of Veterinary Medicine, Institute of Veterinary Anatomy, Histology and Embryology, LMU, Munich, Germany

**Keywords:** Urogenital reproductive disorders, Mechanisms of disease

## Abstract

Salpingitis is a common cause for subfertility and infertility both in humans and animals. However, the effects of salpingitis on tubal function and reproductive success are largely unknown. Therefore we set out to investigate the effects of inflammation on sperm and oocyte transport and gameto-maternal interaction in the oviduct using the bovine as a model. For this purpose, oviducts revealing mild (n = 45), moderate (n = 55) and severe (n = 45) inflammation were obtained from cows immediately after slaughter and investigated by live cell imaging, histochemistry and scanning electron microscopy. Our studies showed that endometritis was always correlated with salpingitis. Moderate and severe inflammation caused a significant increase in the thickness of tubal folds (p < 0.05). Severe inflammation was characterized by luminal accumulations of mucus and glycoproteins, increased apoptosis, loss of tight junctions and shedding of tubal epithelial cells. The mean ciliary beat frequency (CBF) in the ampulla was significantly reduced as compared to the controls (p < 0.05). The higher the grade of inflammation, the lower was the CBF (p < 0.001). In severe inflammation, spermatozoa were stuck in mucus resulting in decreased sperm motility. Our results imply that tubal inflammation impairs proper tubal function and leads to reduced sperm fertilizing capacity.

## Introduction

Salpingitis is defined as an infection and inflammation in the oviducts^1–3^, which are the sites of oocyte and sperm transport, fertilization and early embryonic development^4–10^. Thus, salpingitis is a major cause of subfertility and infertility in humans and animals^11,12^. Tubal inflammation can be acute or chronic and occurs in mild, moderate or severe grade^13^. It is induced by infection, mostly spreading through the lower tract and ascending into the upper genital tract^11,14^. Common ascending inflammatory conditions reported in cows are vulvovaginitis^15^, cervicitis^16^, endometritis^17^, salpingitis^18^ and perioophoritis^19^. Salpingitis^20^ and endometritis^21^ have been reported as the most common bovine genital inflammatory disorder. They are characterized by increased vascularization and secretion as well as marked infiltration of the tubal and/or endometrial tissue with neutrophils and plasma cells^22,23^. In the bovine, the bacteria associated with genital inflammation are mostly *Escherichia coli*, *Trueperella pyogenes*, *Trichomoniasis, Campylobacteriosis, Fusobacterium, Prevotella, bacteroides, Staphylococci* and *Streptococci* species^24–29^. Extensive studies have been carried out and published on symptoms, diagnosis and treatment of endometritis in humans and in animal models^27,30–37^ whereas corresponding studies on salpingitis are scarce. Clinical signs of bovine genital tract inflammation include mucopurulent or purulent vaginal discharge^38^ as well as fever, reduced appetite, tiredness/dullness, no sign of estrus, reduced non-return rate (i.e. no pregnancy) and decreased milk yield. The diagnosis of endometritis in the bovine is done either by ultrasonography or microscopic analyses of (a) cellular material obtained with the cytobrush^39–42^, (b) fluid obtained by uterine flushing^43,44^ or (c) endometrial smears determining polymorphonuclear neutrophils (PMNs), lymphocytes and macrophages^38,39^. However, specific diagnostic tools for bovine salpingitis are lacking as the oviducts are well hidden in the abdomen and cannot be visualized by routine imaging technologies^45,46^.

In the bovine, endometritis and salpingitis cause a huge economic burden in cattle industry by reducing milk yield, conception rates as well as a prolonged breeding season^38,47^. Further to that, cost of treatment was estimated to €1.4 billion in the EU and up to $650 million in the US^48^.

In humans, inflammation of the upper female genital tract (fallopian tubes, ovaries, uterus) including the inside pelvis is collectively known as pelvic inflammatory disease (PID). It is associated with impaired reproductive health and is the most common cause of infertility in females^3,49^. Due to the significant long-term sequelae, widespread health complications and reproductive morbidity, PID is a major public health concern^50–52^. PID is mostly found in sexually active women of reproductive age^11,14,53,54^ and is often correlated with tubal factor infertility (TFI) which accounts for approximately 30–40% of infertility in females^3,55^. As recently shown most cases of TFI are due to salpingitis^56^. Salpingitis occurs after sexually transmitted lower gential infections which are predominantly caused by *Chlamydia trachomatis* and *Neisseria gonorrhoeae*. A study in England estimated that out of 33.6% of women diagnosed with PID, 16.1% had experienced at least an episode of salpingitis^54^. Salpingitis in humans is difficult to diagnose because symptoms are often attenuate or mild. Chronic salpingitis can be asymptomatic, however some acute symptoms include adnexal tenderness, lower abdominal pain and low back pain while some cases of salpingitis are misdiagonsed as acute appendicitis as symptoms are similar^57,58^. In general, laparoscopy is the gold standard to diagnose and confirm the presence of acute salpingitis^45,46^. Salpingitis often causes tubal occlusion and hydrosalpinx^20,21,59^ which might result in ectopic pregnancy^60^.

Thus, it is known that salpingitis has huge effects on reproductive success and fertility both in animals and humans. The precise pathomechanisms, however, are still unclear. Therefore this study aims to provide detailed insights on how inflammation alters tubal morphology and function and how it affects gameto-maternal interaction and successful fertilization. Once these key alterations have been identified this knowledge may open up new concepts for early diagnosis and effective treatment of salpingitis both in animals and humans. Further to that, this knowledge is pivotal to further improve success rates in assisted reproduction in individuals revealing subfertility or infertility.

## Results

### Endometritis is correlated with salpingitis

**Figure 1:**
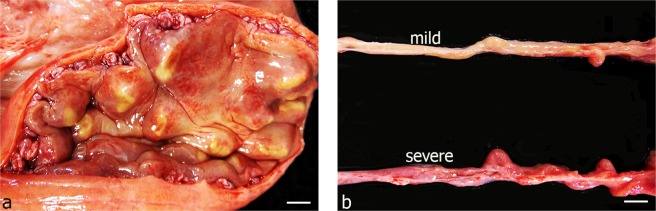
Macroscopic view of bovine uterus and oviduct with inflammation. (**a**) Uterus with severe inflammation (Scalebar: 1.8 cm). (**b**) Bovine left and right oviducts of the same individual showing mild and severe grades of inflammation (Scalebar: 1.0 cm).

A total number of 160 oviducts were analysed in this study (control group n = 15; mild inflammation n = 45; moderate inflammation n = 55; severe inflammation n = 45). The diagnosis and grouping of the oviducts were made based on macroscopic assessment (i.e. increased vascularisation, reddening of mucosa as well as amount and consistency of secretions in oviducts and uterine horns) and were confirmed with pathohistological analyses (number of lymphocytes/0.1 mm^2^). Quantitative analyses revealed that endometritis (Fig. 1a) is always associated with salpingitis (Fig. 1b). 91% of the cows revealed bilateral salpingitis and 9% had unilateral salpingitis. In cows with bilateral salpingitis, 45% of the cows showed the same grade of inflammation on the right and left oviducts of the cows while 55% revealed different grades of inflammation on the right and left oviduct. Figure 1b depicts a right and left oviduct of the same individual revealing mild and severe inflammation, respectively. This highlights the possible occurrence of different grades of inflammation in the oviducts of one individual. In regard to the individuals revealing unilateral salpingitis, 89% of the cows (6/7) revealed inflammation in the right oviduct only, 11% of the cows (1/7) in the left oviduct only.

Inflammation of the ovary or peritoneum was not visible in the individuals investigated.

### Severe inflammation is mostly due to infection with trueperella pyogenes

Microbiological analysis of uterine and oviductal fluid of 14 cows with mild, moderate and severe inflammation revealed that the same bacteria were present both in the uterus and in the oviduct. Cultures obtained from cows with mild and moderate inflammation showed no bacterial growth in culture. In the majority of cows with severe inflammation, Trueperella pyogenes (also known as Actinomyces pyogenes or Arcanobacterium pyogenes) was found which is known to cause purulent infections also in the mammary gland and in the liver. In one individual with severe inflammation Streptococcus pluranimalium was found, in another one Fusobacterium necrophorum was diagnosed.

### Severe inflammation is characterized by increased vascularization and fusion of oviductal folds

**Figure 2:**
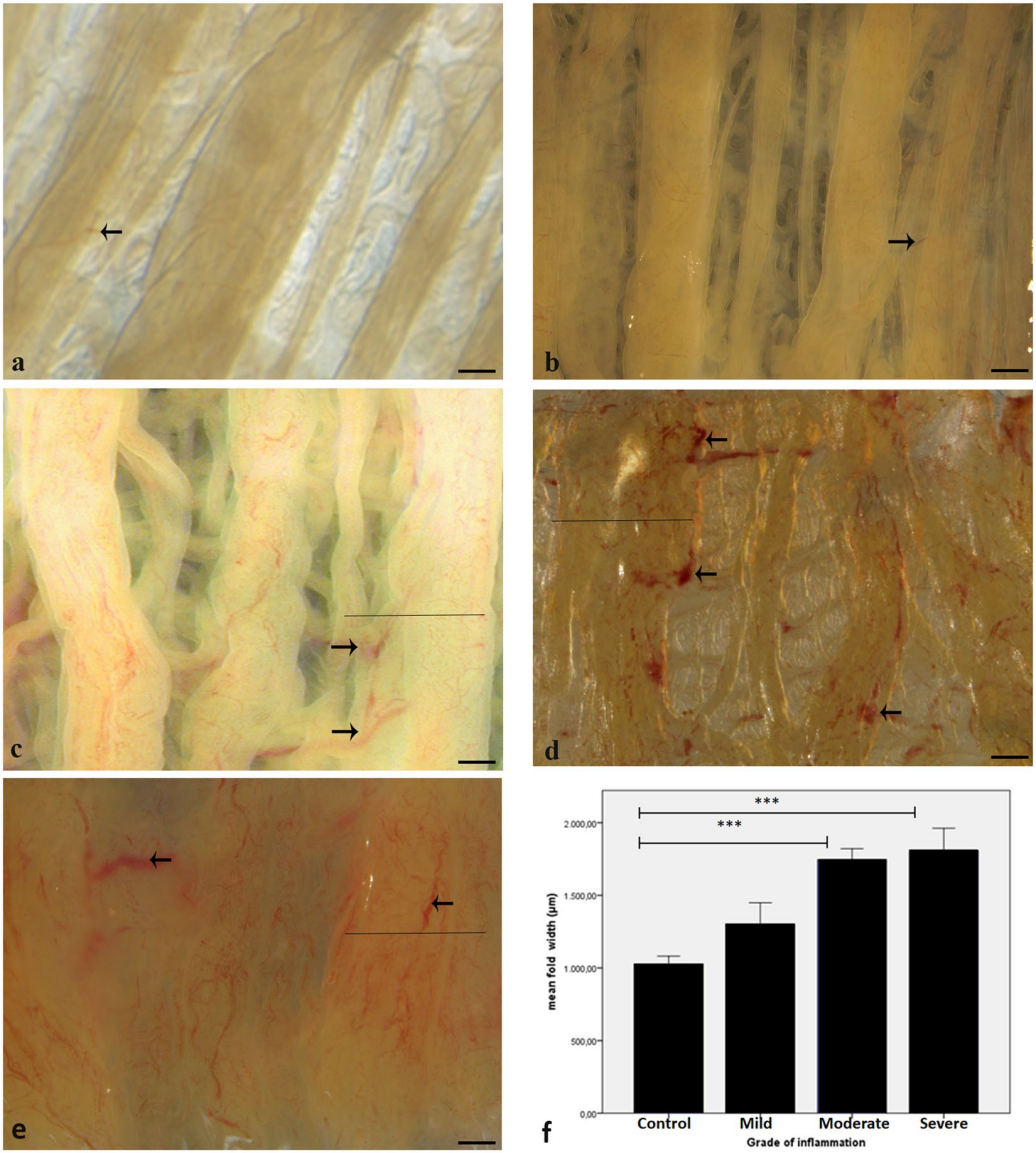
Effects of inflammation on vascularisation and thickness of oviductal folds. Luminal surface of bovine ampulla from (**a**) Healthy control cow. (**b**) Cow with mild inflammation. (**c**) Cow with moderate inflammation. (**d**,**e**) Cow with severe inflammation. In moderate and severe inflammation, the vessels are dilated and clearly visible (**c**–**e**, arrows) causing reddening of the mucosa (**e**). Moderate and severe inflammation are characterized by fusion of primary folds (**c**–**e**, lines) which is not seen in controls (**a**) or in mild inflammation (**b**). (**d**) In moderate and severe inflammation, the thickness of oviductal folds is significantly increased as compared to the healthy controls (ANOVA post hoc Dunnett’s test, ***p < 0.001; Error bar = +/−1 S.E.M) (Scalebars: 600 μm).

In healthy control cows tubal vessels were rarely apparent (Fig. 2a, arrow). There was a slightly increased visibility of vessels in cows with mild inflammation (Fig. 2b, arrow). In cows with moderate (Fig. 2c) and severe (Fig. 2d,e) inflammation, vessels were dilated and clearly visible (arrows) causing reddening of the mucosa (Fig. 2e, arrows). Further to that, inflammation was correlated with fusion of folds in moderate and severe inflammation (Fig. 2c–e, line) which was not seen in controls (Fig. 2a) or in mild inflammation (Fig. 2b). When measuring the largest cross sectional diameter of folds in the ampullae of healthy and inflamed oviducts, the primary folds of the oviducts revealing moderate and severe inflammation were significantly thicker as compared to the healthy control oviducts (ANOVA post hoc Dunnett’s test, ***p < 0.001; error bar = +/−1 SEM, Fig. 2f).

### Salpingitis occurs with increased secretory activity of epithelial cells, ciliary clumping, edema and increased numbers of lymphocytes

**Figure 3:**
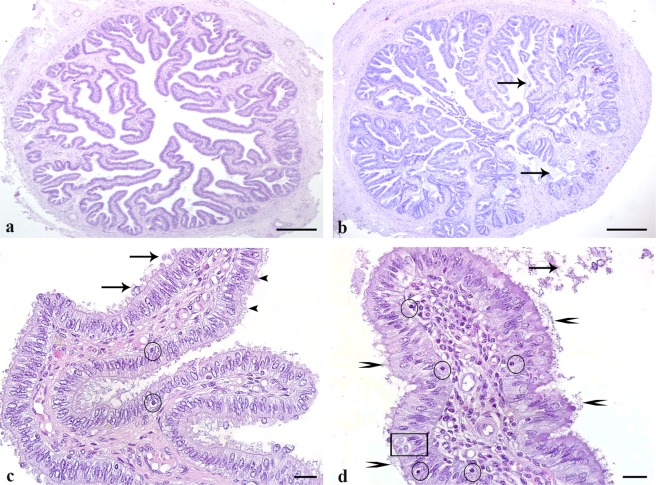
Effects of inflammation on oviductal histomorphology in control animals (**a**) as compared to animals with mild (**c**) and severe inflammation (**b**,**d)**. H&E staining. (**a**) Ampullae of healthy cows reveal a clear lumen. (**b**) In cows with severe inflammation of the oviduct accumulation of cell detritus and secretions are present in the lumen (arrows). (**c**) In mild inflammation, secretory activity of the epithelium is increased (arrows), secretory cells are bulging into the lumen (arrowheads) and few lymphocytes are visible (circle) (**d**) Severe inflammation is characterized by mucus accumulation (arrows), ciliary clumping (arrowheads) and edema which are not seen in the control. Apototic cells are seen frequently (quadrate). Numbers of lymphocytes both in the epithelium and in the connective tissue are distinctly increased (circles). (Scalebars: (**a**,**b**) 200 µm, (**c**,**d**) 20 µm).

Control oviducts regularly showed a clear lumen (Fig. 3a). Contrary oviducts revealing severe inflammation revealed high amounts of secretions and cell detritus in the lumen (Fig. 3b,d arrows). In mild inflammation slightly increased secretory activity of the epithelium was visible with small amounts of mucus covering the epithelium (Fig. 3c, arrows). In moderate inflammation secretions were not only covering the epithelium but were also present in the lumen. In all grades of inflammation high numbers of secretory cells bulging into the lumen were seen (Fig. 3c, arrowheads). The number of lymphocytes within the epithelium and connective tissue was regularly 0–2 lymphocytes/0.1 mm^2^ in healthy controls. It ranged from 3–5 lymphocytes/0.1 mm^2^ in mild inflammation (Fig. 3c, circle). Oviducts with moderate inflammation revealed 6 to 9 lymphocytes/0.1 mm^2^. Oviducts with severe inflammation showed 10 or more lymphocytes/0.1 mm^2^ (Fig. 3d, circles). Oviducts with severe inflammation revealed huge luminal accumulations of secretions on the top of the epithelium (Fig. 3d, arrows) which contained accumulations of neutrophil granulocytes. Accumulation of secretions near the luminal surface resulted in the clumping of cilia (Fig. 3d, arrowheads). In addition to that, increased numbers of apoptotic cells in the epithelium (Fig. 3d, quadrate) as well as edema characterised by increased space between the collagen fibers in the connective tissue were seen (Fig. 3d). These features were also visible in moderate inflammation, but to a lesser extent.

### Salpingitis leads to accumulation of glycoproteins and acidic mucopolysaccharides

**Figure 4:**
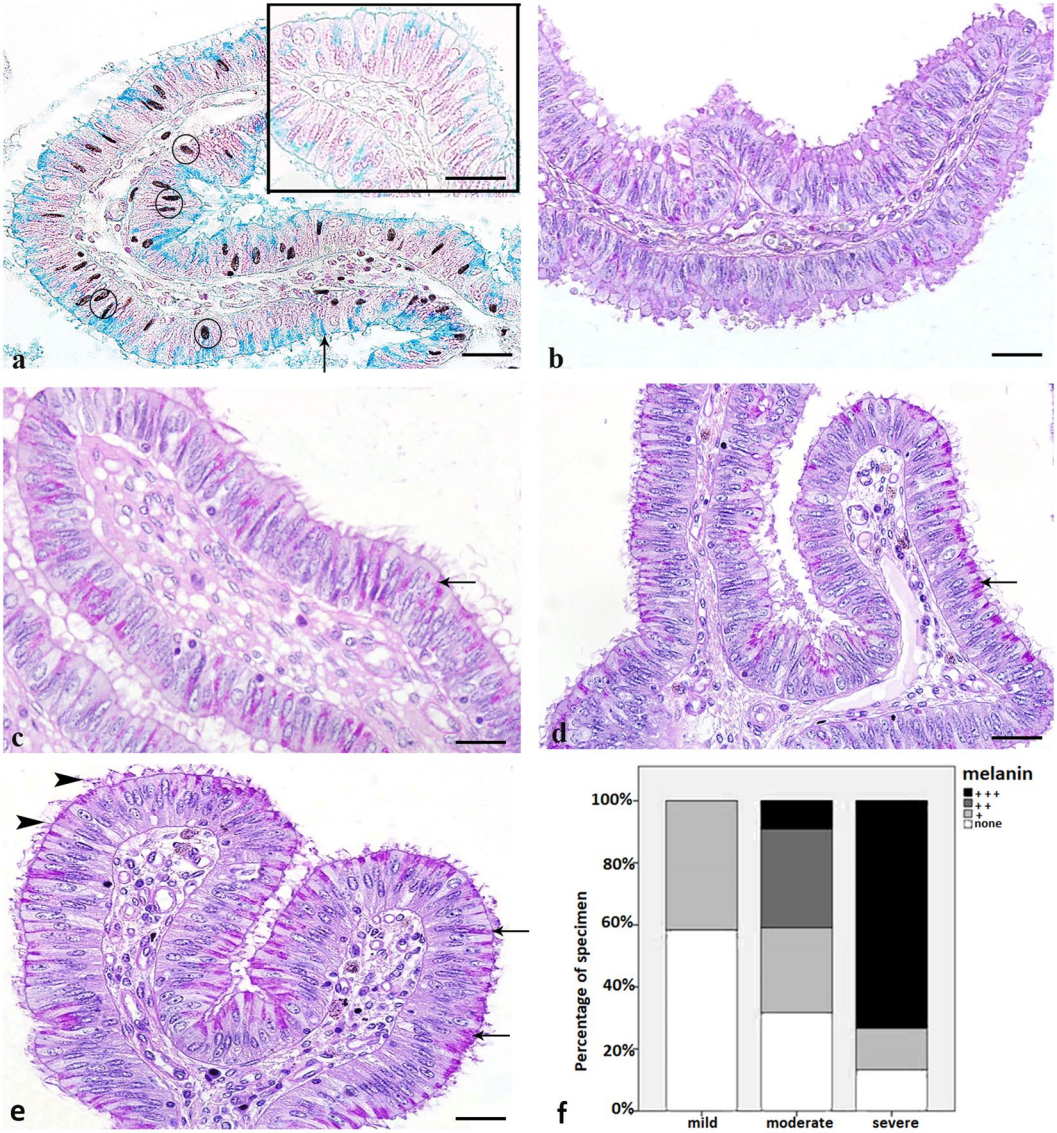
Effects of inflammation on synthesis of acidic mucopolysaccharides (Alcianblue) staining and glycoproteins (PAS) staining in the bovine ampulla. (**a**) Synthesis of acidic mucopolysaccharides is distinctly increased in ciliated and secretory cells from cows with severe inflammation as compared to controls (inlay). Peg cells also stain positive (arrow). In chronic severe inflammation melanin accumulation is seen in nuclei of the epithelium and the connective tissue (circles). (**b–e**) PAS staining in a control oviduct (**b**) and oviducts with mild (**c**), moderate (**d**) and severe (**e**) inflammation. Synthesis of glycoproteins is distinctly increased in oviducts with moderate and severe tubal inflammation (**d**,**e**) as compared to control oviducts (**b**) and oviducts with mild inflammation (**c**,**d**) The accumulation of melanin is significantly increased in oviducts from cows with severe inflammation as compared to oviducts from cows with mild inflammation (Goodman and Kruskal’s gamma = 0.701, p < 0.001). (Scalebars: 15 µm).

Alcianblue staining (pH 2.5) revealed an increased synthesis of acidic mucopolysaccharides in the apical parts of ciliated cells of cows with moderate and severe inflammation (Fig. 4a, blue staining) as compared to the healthy controls (Fig. 4a, inlay). In addition to that peg cells which have already secreted into the lumen stained positive for Alcianblue (Fig. 4a, arrow). Further to that, Periodic Acid Schiff Reaction demonstrated an increase in synthesis of glycoproteins in moderately (Fig. 4d) and severely (Fig. 4e) inflamed oviducts as compared to the healthy control oviducts (Fig. 4b) and mildly inflamed oviducts (Fig. 4c). Glycoproteins were regularly localized in the apical parts of the secretory and ciliated cells (Fig. 4c–e, arrows) as well as in the mucus in the lumen and in the mucus covering the cilia (Fig. 4e, arrowheads). Amylase digestion confirmed that the pink staining was due to the presence of glycoproteins and not to glycogen. Thus, glycogen storage in the tubal epithelial cells was absent in healthy control cows during diestrus as well as in individuals with inflammation.

### Chronic severe inflammation is characterized by accumulation of melanin

Accumulations of black and brown pigments were seen in the nuclei of ciliated cells, the nuclei of basal cells as well as in fibroblasts and fibrocytes of the underlying connective tissue (Fig. 4a, circles). Treatment with H_2_O_2_ confirmed that the pigment was melanin and not hemosiderin. The highest amount of melanin was seen in oviducts with severe inflammation (Fig. 4a). Accumulation of melanin occurred in 35% of all cows with salpingitis. Whereas in acute severe inflammation large numbers of granulocytes were seen, chronic inflammation was characterized by the predominance of mononuclear cells. Accumulation of melanin occurred predominantly in individuals with predominance of mononuclear cells, i.e. in individuals with chronic inflammation. The accumulation of melanin was significantly increased in oviducts from cows with severe inflammation as compared to oviducts from cows with mild inflammation (Goodman and Kruskal’s gamma = 0.701, p < 0.001, Fig. 4d).

### Severe inflammation results in deciliation, apoptosis and loss of tight junctions

**Figure 5:**
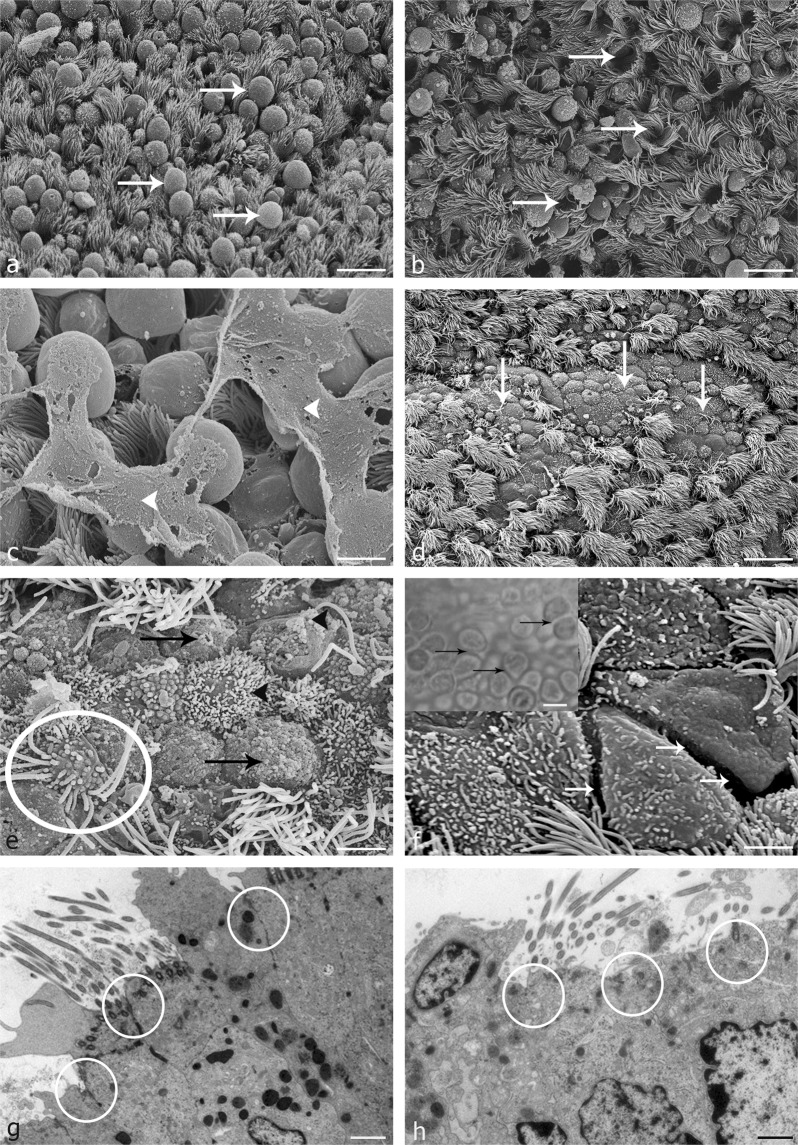
Effects of inflammation on oviductal microarchitecture as seen by scanning electron microscopy (SEM) and transmission electron microscopy (TEM). (**a**) All grades of inflammation (here mild inflammation) lead to increased secretory activity in the ampullar epithelium with bulging cells being prominent (arrows). (**b**) Empty spaces occur after secretory cells leave the epithelium. (**c**) In moderate and severe inflammation, layers of mucus are covering the cells (arrowheads). (**d**) Severe inflammation is characterized by deciliation of tubal cells (arrows). (**e**) Cilia show irregular length, loss of uniformity and breakdown (circle). Further to that, alterations in density and uniformity of microvilli of the secretory cells occur (arrow). (**f**) Severe inflammation results in loss of tight junctions which can also be seen in live cell imaging (inlay, circle, see Video 1). (**g**,**h**) Loss of tight junction is seen in some areas (**h**, circles), areas with intact tight junctions are also visible (**g**, circles). (Scalebars: (**a**,**b)** 20 µm; (**c**) 7 µm, (**d**) 25 µm, (**e**) 5 µm, (**f**–**h**) 3.5 µm, Inlay: 8 µm).

**Figure 6:**
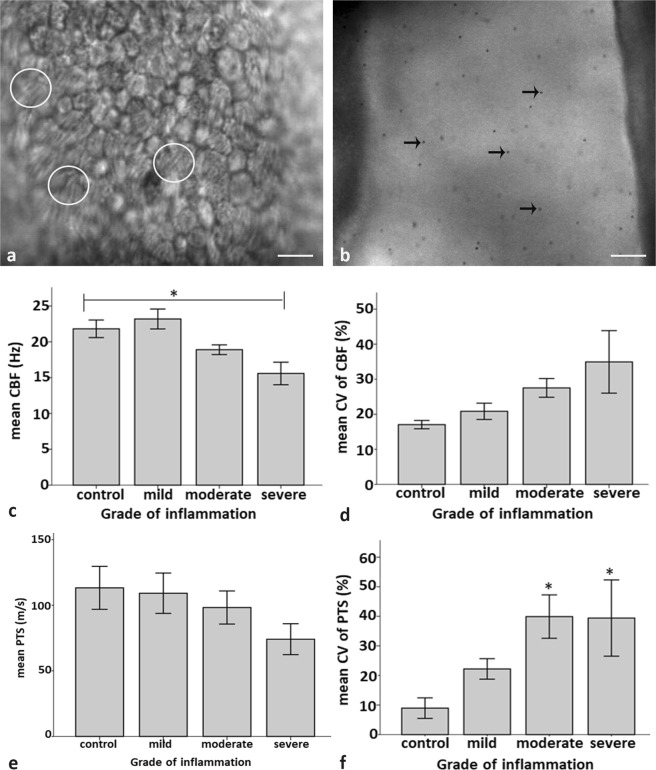
Effects of inflammation on oviductal ciliary beat frequency (CBF) and particle transport speed (PTS). (**a**) CBF is calculated by analysing the differences of shades of grey during the beat of the cilia of single cells (circle, see Video 2). (**b**) PTS is calculated by tracking polystyrene dynabeads when moving between the tubal folds (arrows, see Video 3). (**c**) In severe inflammation the mean CBF is significantly decreased (see Video 1) as compared to the controls (see Video 2, Post hoc Bonferroni test, p = 0.04). (**d**) The higher the grade of inflammation, the higher is the mean coefficient of variation of CBF (correlation coefficient Spearman rho = 0.441, p = 0.001). (**e**) The mean PTS (see Video 3) is not significantly different in controls and inflamed oviducts. (**f**) However, the mean coefficient of variation of PTS is significantly higher in oviducts with moderate and severe inflammation (see Video 4) as compared to control oviducts (Post hoc Bonferroni test, p = 0.038 and 0.033, respectively.) Error bar = +/−1 S.E.M. (Scalebars: (**a**) 20 µm, (**b**) 50 µm).

In all grades of inflammation high numbers of secretory cells bulging into the lumen were visible (Fig. 5a, arrows). These cells later leave the epithelium leaving empty spaces behind (Fig. 5b, arrows). In mild inflammation predominance of bulging cells were the most obvious characteristic feature of the epithelium (Fig. 5a, arrows). In moderate and severe inflammation additionally, layers of mucus were covering the tubal cells (Fig. 5c, arrowheads). Further to that, severe inflammation was characterized by wide areas revealing deciliation (Fig. 5d, arrows) as well as irregular lengths and loss of uniformity and breakdown of cilia (Fig. 5e, circle). Additionally, alterations in density and uniformity of microvilli covering the secretory cells (Fig. 5e, arrow) as well as a high variability in form and size of epithelial cells (Fig. 5e) were visible. Further to that, scanning electron microscopy revealed cell demarcation and loss of tight junctions in severe inflammation (Fig. 5f, arrows) which was also seen in live cell imaging (Fig. 5f, inlay, arrows, see Video 1). The arrangement of cells is clearly impaired as compared to a healthy control (Fig. 6a). As confirmed with transmission electron microscopy, loss of tight junctions in severe inflammation occurred in some areas of the ampullar epithelium (Fig. 5h, circles). However, areas with proper tight junctions between the cells were also present (Fig. 5g, circles).

### Severe salpingitis results in reduced ciliary beat frequency (CBF)

CBF was calculated as differences in the shades of grey which occur during ciliary beating (Fig. 6a, see Video 2, healthy control). CBF was significantly decreased in inflamed oviducts (Fig. 5f, inlay, see Video 1) as compared to the healthy controls (linear mixed model, p < 0.001, see Video 2). CBF was significantly decreased when comparing severe inflammation (see Fig. 5f, inlay, Video 1) with controls (see Video 2, Fig. 6c, Bonferroni post hoc test, p = 0.004). However, when comparing mild and moderate inflammation with controls, the decrease in CBF was not significant (Fig. 6c, p = 1.000 and p = 0.350, respectively). The higher the grade of inflammation, the lower was the mean CBF (correlation coefficient Spearman rho = −0.516, p < 0.001). In regard to the mean coefficient of variation of CBF, it was not significantly altered in inflamed oviducts as compared to healthy controls (linear mixed model, p = 0.104) or when comparing control versus mild, moderate and severe inflammation (Fig. 6d, Bonferroni post hoc test, p = 1.000, p = 0.618 and p = 0.069, respectively). However, the higher the grade of inflammation, the higher was the mean coefficient of variation of CBF (correlation coefficient Spearman rho = 0.441, p = 0.001, Fig. 6d).

Further to that particle transport speed (PTS) relating to oocyte and early embryo transport speed was measured by tracking polystyrene dynabeads moving within the oviduct (Fig. 6b, see Video 3, healthy control). The overall mean PTS in inflamed oviducts was not significantly altered in inflamed oviducts as compared to healthy oviducts (linear mixed model, p = 0.258). Similarly there were no significant differences when comparing the PTS in healthy control oviducts with oviducts revealing mild, moderate or severe inflammation (Fig. 6e, Bonferroni post hoc test, p = 1.000, p = 0.542, p = 0.249). However, the higher the grade of inflammation the lower was the PTS (correlation coefficient Spearman rho = −0.29, p = 0.050, Fig. 6e, see oviduct with severe inflammation, Video 4). Further to that, the degree of inflammation had a significant influence on the mean coefficient of variation of PTS. Thus, the mean coefficient of variation was significantly higher in inflamed oviducts as compared to healthy controls (mixed linear model, p = 0.027). When comparing the mean coefficient of variation of PTS in healthy oviducts with that of oviducts with mild, moderate or severe inflammation, a significant increase was seen in individuals with moderate and severe inflammation (p = 0.038 and p = 0.033, respectively). The higher the grade of inflammation, the higher was the mean coefficient of variation seen (correlation coefficient Spearman rho = 0.525, p < 0.001, Fig. 6f).

### Salpingitis decreases sperm motility in the tubal sperm reservoir

**Figure 7:**
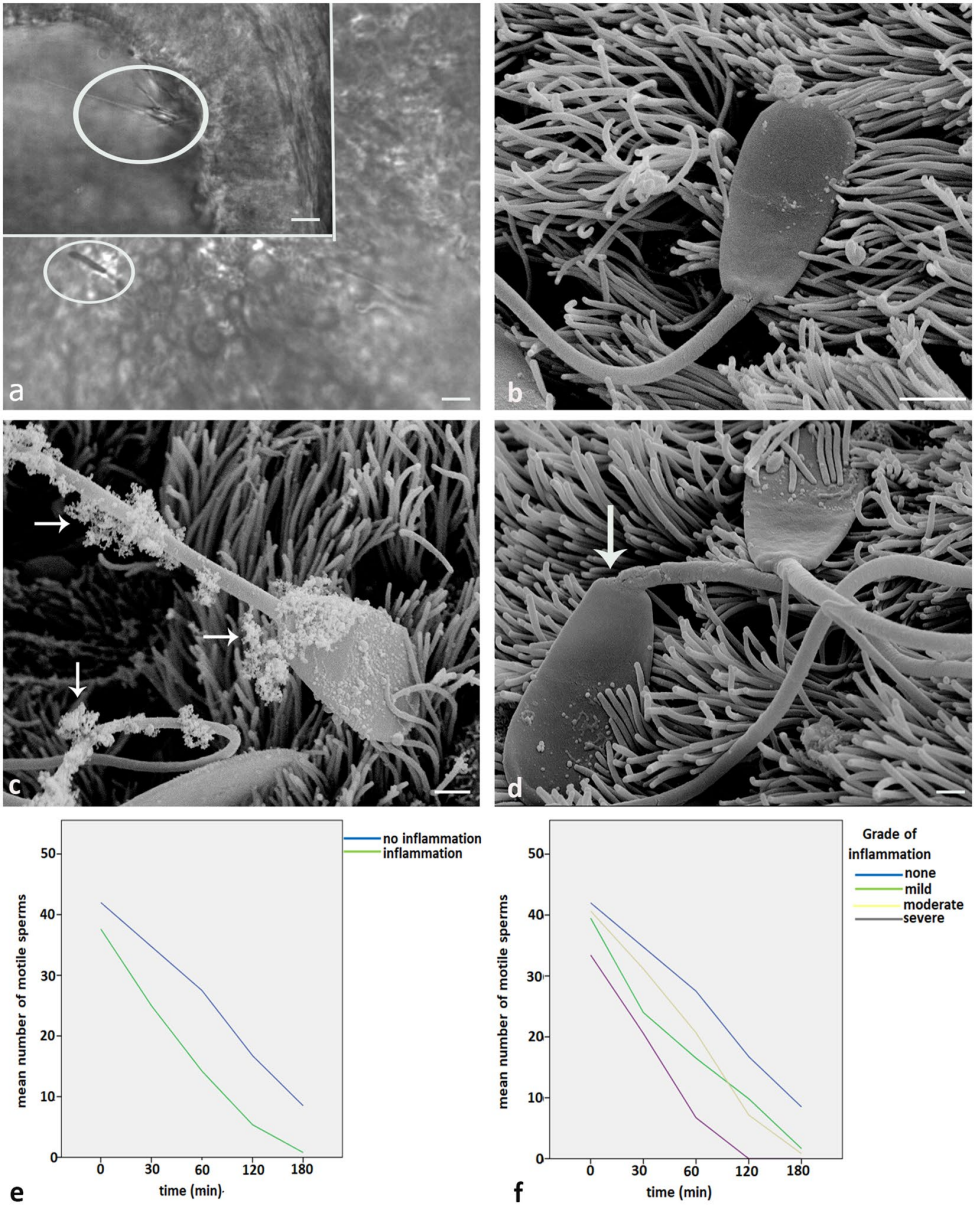
Effects of inflammation on bound sperm motility and survival time of sperm within the oviduct. (**a**) In healthy control oviducts sperm are bound to the cilia of the ciliated cells in a tangential angle (Inlay, see Video 5). Contrary in inflamed oviducts, the majority of sperm lie flat on the epithelium (see Video 6). (**b**) A sperm lying flat in an oviduct with moderate inflammation as seen by SEM. (**c**) In severe inflammation, head and tail of spermatozoa are covered with mucus. (**d**) In inflamed oviducts a lot of sperm show incorrect tail insertions on the neck (arrow). (**e**) The mean number of motile sperm in cows with tubal inflammation is significantly decreased as compared to the controls. (Area under the curve, two-sided T test for independent samples). (**f**) The mean number of motile bound sperm in cows with severe inflammation is significantly decreased as compared to the controls (Area under the curve, two-sided T test for independent samples). In severe inflammation the average time of sperm with active tail movement being present is reduced to 2 hrs (Scalebars 3 µm).

In healthy control oviducts spermatozoa are bound to the cilia of the ciliated cells in a tangential angle (Fig. 7a, Inlay, see Video 5). Contrary in inflamed oviducts the majority of sperm lie flat on the epithelium (Fig. 7a,b), with their head and tail being stuck in the mucus (Fig. 7c, arrows, see Video 6).

Furthermore, due to being stuck in mucus, most sperm revealed incorrect tail insertions on the neck (Fig. 7d, arrow). Overall, the mean number of motile sperm bound to the ciliated cells in the oviduct was significantly decreased in cows revealing salpingitis as compared to healthy controls (Fig. 7e). The statistical analysis was done by comparison of the area under the curve (AUC). The D’Agostino Pearson test accepted normality for both groups with and without inflammation (Fig. 7e, two sided t-test for independent samples, p = 0.007). When comparing the mean number of motile sperm in the oviduct in healthy individuals with those in individuals with mild, moderate and severe inflammation the mean number of motile sperm was significantly lower in cows with severe inflammation (comparison of the area under the curve, D’Agostino Pearson test accepted normality for all 4 groups, two sided t-test for independent samples, p = 0.00,1 Fig. 7f).

Further to that, the average time of sperm with active tail movement being present in mildly and moderately inflamed oviducts was 3 hs as compared to 4 hs in healthy oviducts (Fig. 7e). In cows with severe inflammation, the maximum time of sperm with active tail movement being present was 2 hs (Fig. 7f).

## Discussion

Our study is the first comprehensive description of the effects of inflammation on sperm-oviduct interaction in the bovine oviducts. According to our results endometritis is always correlated with salpingitis. Our study provides evidence that salpingitis drastically impairs sperm-oviduct interaction leading to compromised fertilization and reduced fertility. In inflamed oviducts, sperm survival time is reduced and the mean number of motile sperm in the sperm reservoir is significantly decreased in cows with salpingitis as compared to healthy cows. To date, this is the first study describing the effect of inflammation on sperm-oviduct interaction under near *in vivo* conditions using a newly established digital videomicroscopic imaging technology. Most publications on the effect of inflammation on sperm have been performed under *in vitro* conditions using the lipopolysaccharide (LPS) which is derived from gram-negative bacteria such as *Escherichia coli*. LPS has been shown to induce inflammation so that it can be used as valuable model for studying the mechanisms involved in inflammatory diseases of the reproductive tract^61^. To date it has been shown that in rabbits intracervical application of LPS resulted in fewer spermatozoa reaching the oviducts as compared to intraperitoneal application of LPS. This points to the fact that inflammation of the genital tract decreases the effectivity of sperm transport. As a consequence fewer sperm reach the site of fertilization. Our results provide first evidence that even if the sperm manage to reach the oviduct they have reduced vitality, survival time and decreased fertilizing capacity. This is supported by the findings of O’Doherty *et al*. (2016) who found reduced bound sperm motility in the bovine tubal reservoir after incubation with LPS^62^. The negative effects of LPS on sperm motility and sperm storage have also been reported in rabbits^63^ and in Teleogryllus oceanicus^64^. In addition to that, our study revealed that spermatozoa in inflamed oviducts often reveal damaged plasma membranes. This might be one of the predominant causes for reduced sperm survival time in inflamed oviducts. On the plasma membrane of the head, species-specific lectins are located which mediate the sperm binding to the sugars of the cilia^65–68^. As a consequence, damage to the plasma membrane might result in altered function of these lectins resulting in compromised initiation of the signal cascades which are induced after sperm binding to the tubal epithelium. This might be the cause for loss of viability and motility in the sperm reservoir in salpingitis. This negative impact on sperm fertilizing capacity is increased by the fact that sperm in inflamed oviducts are stuck in mucus preventing them to detach after ovulation has occurred. Detachment of sperm is achieved by hyperactivation which is characterized by asymmetrical flagellar bending with a high amplitude^67^. Hyperactivation is not only pivotal for detachment of spermatozoa but also supports the migration of the sperm through the oviduct and enables the penetration of the sperm through the cumulus oophorus^69^. All these processes (sperm hyperactivation, detachment, migration and penetration of the cumulus oophorus) are compromised by accumulation of mucus on the head, neck and tail of sperm which were seen in excessive amounts in our live cell imaging and scanning electron microscopic studies. In addition to that, sticking of sperm in the mucus was seen to induce sperm breaks in the neck region. The molecular actions of naturally occurring inflammation in the female genital tract on sperm functional integrity have not been reported yet. However, in *in vitro* studies, the mechanisms of sperm damage by LPS have been attributed to mitochondrial dysfunction and activation of oxidative phosphorylation in boars^70^, cytokine release in rabbits^63^ as well as to reduction of intracellular cAMP levels in humans^71^. Survival of the sperm is further compromised by morphological alterations of the ampulla induced by inflammation. As seen in stereomicroscopic analysis, inflammation causes significant increases in the width of the folds of the bovine ampulla – mostly as a consequence of fusion of mucosal folds. The microarchitecture of the oviductal folds greatly affects the transport of sperm as tubal patency is compromised^72^. However, successful fertilization can only occur in a patent oviduct where a precisely timed transport is maintained^5,73^. Further to that, fused and thickened epithelial folds may inhibit the normal action of smooth muscle contraction thus further impairing gamete transport. Patency of the oviduct in moderately and severely inflamed oviducts is distinctly affected by increased secretory activity and accumulation of mucus in the lumen. Histochemical analyses of inflamed oviducts confirmed that the secretions in inflamed oviducts contain increased amounts of glycoproteins and acidic mucopolysaccharides as compared to healthy oviducts. As a consequence, transport of the gametes and the early embryo in inflamed oviducts is not only mechanically impaired by accumulation of mucus, but also functionally compromised by altering ciliary beat frequency in the oviduct. Thus, ciliary beat frequency is significantly decreased in severe inflammation as compared to the controls. According to our SEM studies this is not only due to the accumulation of mucus on the cilia inhibiting movement but also areas of deciliation within the mucosa which is particularly seen in severe inflammation. In contrast to our results O’Doherty *et al*., (2016) found an increased CBF in the bovine after addition of LPS^62^. This might be due to the fact that natural inflammation is accompanied by massive accumulation of mucus and inflammatory molecules which are absent in LPS treatment only. In addition to that, it might be hypothesized that in the beginning of bacterial invasion, the oviduct initially seeks to increase tubal clearance by increasing ciliary beat frequency which is abandoned later. Our studies showed, for the first time, that accumulation of melanin is an indicator of chronic inflammation in the oviduct. Thus, the amount of melanin in the nuclei of epithelial cells in severe inflammation is significantly increased as compared to mild and moderate inflammation. Accumulation of melanin is controlled by melanin concentrating hormone (MCH). Studies suggest that MCH modulates inflammatory responses via activation of its receptor MCHR1 in various cell types^74,75^. This is supported by the report by Ziogas *et al*. (2014) who identified colonic epithelial cells as targets of MCH in the intestine^76^. Studies have also reported on the proinflammatory role of MCH. This was seen in intestinal inflammatory study carried out in mice. Mice treated with alpha-MSH attenuated experimental colitis while mice deficient for the alpha-MSH receptor MC1R developed aggravated DSS-induced colitis. Thus, the accumulation of melanin might be used as valuable tool for the diagnosis of chronic inflammation in the oviduct.

In summary, our study is the first to demonstrate that salpingitis distinctly impairs gameto-maternal interaction. In inflamed oviducts, sperm survival time and sperm motility in the sperm reservoir are decreased. Massive accumulation of mucus on head, neck and tail compromise hyperactivation, detachment and migration of sperm after ovulation. Due to increased synthesis of glycoproteins and acidic mucopolysaccharides both transport and nutrition of the gametes and the early embryo are negatively affected. This is multiplied by morphological and functional alterations in the tubal epithelium which is characterized by accumulation of mucus and cell debris, abundant amounts of secretory cells leaving the epithelium, loss of cellular uniformity, increased apoptosis and loss of tight junctions.

These results imply that evaluating the presence of melanin, the epithelial secretory activity and the integrity of tight junctions might be valuable tools for the precise diagnosis of inflammation in the oviducts. As a novel therapeutic concept, maintaining tubal patency and decreasing accumulation of cell debris and mucus by tubal flushing might be a valuable tool for increasing fertility in diseases which come along with inflammation. These new insights are pivotal for further increasing the success rates of subfertility and infertility treatment and for improving the outcomes of assisted reproductive technologies (ART) both in humans and animals.

## Materials and Methods

### Ethics

This study did not require ethical approval as bovine tissues were obtained from the abattoir after slaughter. Full ethical exemption was granted by the Office of Research Ethics, University College Dublin (AREC-E-17-07-Kölle).

### Tissue collection

Bovine oviducts from cows revealing mild (n = 45), moderate (n = 55) or severe (n = 45) inflammation were obtained from the abattoir immediately after slaughter and compared to specimens obtained from healthy cows (n = 15). Experiments were performed within two hours of collection. The cycle stage was determined by macroscopic assessment of the ovary, uterus and cervix. All specimens used for this study were from cows in diestrus. Each uterine horn and oviduct was examined macroscopically to determine the presence of inflammation in the tract.

The precise criteria to discriminate between mild, moderate and severe inflammation were as following: (a) vascularisation (mild: – to +, moderate: ++ and severe: +++), (b) colour of the endometrium and tubal mucosa (mild: slight red, moderate: distinctly red and severe: dark red) and (c) amount and composition of secretions (mild: +, moderate: ++: predominantly watery secretions and severe: +++: viscous secretions, often with pus). The grouping was further confirmed by pathohistological analysis i.e. number of lymphocytes present in 5 regions of interests (ROIs) (mild: 3–5 lymphocytes/0.1 mm^2^, moderate: 6–9 lymphocytes/ 0.1 mm^2^ and severe: 10 or more lymphocytes/0.1 mm^2^).

### Microbiological analysis

For microbiological analyses, uterine horns and oviducts showing mild, moderate and severe inflammation were investigated (n = 14). Endometrial and tubal fluids were collected by flushing with 100 µl sterile PBS and collecting the fluid into sterile Eppendorf tubes (Eppendorf, Germany). Samples were snap frozen in liquid nitrogen and stored at −20 °C. After thawing, routine microbiological analyses were performed at the Institute for Infectious Diseases and Zoonoses, Ludwig Maximilian University Munich, Germany. For determination of aerobic bacteria Mueller Hinton agar plates (#97580 Sigma Aldrich, Steinheim, Germany) with 5% sheep-blood were used. For diagnosis of anaerobic bacteria Schaedler agar plates (#212189 Becton Dickinson Heidelberg, Germany) with 5% sheep blood were applied. The results were recorded in a semi-quantitative way for every colony type as follows: after enrichment only; (+)  = fewer than 10; + = 10–20; ++ 21–40; +++ >40 colony-forming unit (cfu)/plate. Identification of every colony type was performed using a Matrix-Assisted-Laser-Desorption/Ionisation Time of Flight Mass Spectrometer (Microflex LT and MALDI Biotyper Identification-Software Version 3.1, Bruker Daltonik GmbH, Bremen, Germany).

### Sperm preparation

Frozen bull sperm were obtained from the Irish Cattle Breeding Centre, Kildare, Ireland and the Cattle Breeding Centre Schleswig Holstein, Germany (breed: Holstein-Friesian, age 1–3 years). Sperm were thawed by immersion in a water bath at 39 °C for 10 sec. Sperm were then washed in 1.5 ml of a modified Tyrode balanced salt solution (Sperm TALP: 99 mM NaCl, 3.1 mM KCl, 25 mM NaHCO_3_, 0.4 mM NaH_2_PO_4_, 1.1 mM MgCl_2_, 2 mM CaCl_2_, 10 mM HEPES, 1 mM pyruvate, 25.4 mM lactate (pH 7.45, 290–300 mOsm/kg) to remove the semen diluent. After centrifugation the supernatant was removed and the pellet was resuspended in 100 μl of pre-warmed Sperm Talp buffer. Post wash motility was assessed using an Olumpus phase contrast inverted microscope (Olympus VKC41SF, Germany) using the lens LCAch PHP N20x/0.4. Only sperm with a post-wash motility of 60% were used for experiments.

### Stereomicroscopy

1 cm pieces of bovine ampulla were freed from connective tissue and were cut open and placed in a petri dish. Tubal folds and vascularisation were imaged in oviducts with mild (n = 5), moderate (n = 5) and severe (n = 5) inflammation (n = 5) as well as in controls (n = 5) using the Olympus DP- SZX12 microscope and Olympus U-TV1X-2 camera (Tokyo, Japan). The largest cross sectional diameter of primary folds ampulla was measured in oviducts of all grades of inflammation and compared to healthy controls using Image J (U.S. National Institutes of Health; https://imagej.nih.gov/ij/).

### Histomorphological analysis

The middle third of the ampulla (1 cm) from inflamed (n = 30) and healthy (n = 12) oviducts was freed from connective tissue and fixed in Bouin’s fixative for 22 hrs. After washing twice in 70% ethanol and dehydratation in ascending series of ethanols (70%, 80%, 90%, 100% and 100%; 10 mins each, RT) specimens were embedded in paraffin. Sections (6 μm) were cut (microtome: Reichert Jung 2030, Leica Microsystems, Germany), mounted on Superfrost Plus slides (Thermo Scientific, Dublin, Ireland) and stained with H&E. After staining specimens were mounted with DPX (Sigma-Aldrich, Wicklow, Ireland) and coverslipped. Analyses were performed with an Olympus BX51 microscope equipped with an Olympus DP71 camera (Olympus Corp., Tokyo, Japan).

### Histochemistry

Periodic Acid Schiff reaction (PAS) reaction was applied to compare the synthesis of glycoproteins and glycogen in oviducts with different grades of inflammation (n = 30) as compared to healthy controls (n = 10). Amylase digestion was applied in order to discriminate between glycoproteins and glycogens. Sections were dewaxed in xylene (Sigma-Aldrich, Wicklow, Ireland, 3 × 10 mins, RT), followed by rehydration in descending series of ethanols (100%, 100%, 90%, 80% and 70%; 5 mins RT) and treatment with distilled water (5 mins, RT). In the following, specimens were placed in 0.25% periodic acid (Sigma-Aldrich, Wicklow, Ireland; 3.5 mins, RT), running water (10 mins, RT) and distilled water (dipping, RT). Then the slides were placed in Schiff’s reagent (Sigma-Aldrich, Wicklow, Ireland; 5 mins, RT), sulfite water (0.5 g sodium disulfate, 99 ml distilled water, 1 ml 25% hydrochloric acid [HCL]; Sigma-Aldrich, Wicklow, Ireland; RT, 2 × 5 mins, RT) and running water (10 mins, RT). Counterstaining was done with hematoxylin (VWR, Dublin, Ireland; 30 secs, RT). After washing in running tap water (10 mins, RT), sections were dehydrated in ascending series of ethanols (70%, 80%, 90%, 100% and 100%; 5 mins, RT), cleared in xylene (Sigma-Aldrich, Wicklow, Ireland; 3 × 10 mins, RT), mounted with DPX (Sigma-Aldrich, Wicklow, Ireland) and coverslipped. For glycoproteins and glycogen discrimination, slides were pretreated with amylase (Sigma-Aldrich, Wicklow, Ireland; 10 mins, 37 °C) before incubation in periodic acid. All images were taken with an Olympus BX51 microscope equipped with an Olympus DP71 camera (Olympus Corp., Tokyo, Japan).

Alcianblue staining (pH 2.5) was applied to compare synthesis of acidic mucopolysaccharides in inflamed oviducts (n = 30) as compared to healthy control oviducts (n = 10). Sections were dewaxed in xylene (Sigma-Aldrich, Wicklow, Ireland; 3 × 10 mins, RT) and rehydrated in descending series of ethanol solutions (100%, 100%, 90%, 80% and 70%; 5 mins, RT). After washing in distilled water (5 mins, RT) sections were placed in 3% acetic acid (Sigma-Aldrich, Wicklow, Ireland 20 mins, RT). After dipping in distilled water, sections were incubated in Alcianblue solution (2 g alcian blue 8GX; Sigma-Aldrich, Wicklow, Ireland, dissolved in 200 ml of 3% acetic acid pH 2.5; Sigma-Aldrich, Wicklow, Ireland, 30 mins, RT). Slides were then counterstained with 0.1% nuclear Fast Red (Sigma-Aldrich, Wicklow, Ireland; 3 mins, RT). After washing in distilled water, specimens were dehydrated in ascending series of ethanols (70%, 80%, 90%, 100% and 100%; 5 mins, RT) and cleared in xylene (Sigma-Aldrich, Wicklow, Ireland; 3 × 10 mins, RT). Samples were mounted with Dako® (Sigma Chemical, Dublin, Ireland) and coverslipped. All images were taken with an Olympus BX51 microscope equipped with an Olympus DP71 camera (Olympus Corp., Tokyo, Japan). To confirm that the black staining seen in tubal epithelial nuclei was due to accumulation of melanin, specimens were dewaxed and treated with H_2_O_2_ (RT, 5 mins). Whereas melanin does bleach after this treatment, hemosiderin does not bleach.

### Scanning electron microscopy (SEM)

1 cm pieces of the ampulla were obtained from bovine female genital tracts revealing mild, moderate and severe inflammation (n = 30) as well as from healthy controls (n = 10). For analysis of sperm-oviduct interaction, 1 cm pieces of ampulla were co-incubated with 600,000 sperm (30 µl) at 37 °C for 10 mins. All samples were washed in Soerenson’s buffer (1:5 solution of 0.07 KH_2_PO_4_ and 0.07 M Na_2_HPO_4_.2H_2_O, pH 7.4) twice and fixed in 1% glutaraldehyde in Soerensen’s buffer (24 hrs, 4 °C). After washing in Soerenson’s buffer, specimens were dehydrated in series of ascending acetones (10%, 20%, 30%, 40%, 50% and 60% 2×, 5 min; 70%, 80% and 90%, 60 mins; 100%, 12 hrs, RT). The samples were dried at critical point with liquid CO_2_ using the union point dryer CPD 030 (Bal-Tec, Walluf, Germany). Specimens were then coated with 12-nm gold-palladium using the Union SCD 040 sputtering device (Bal-Tec, Walluf, Germany). Samples were analysed using a DSM 950 (Zeiss, Oberkochen, Germany) at magnifications from x50 to x8000 equipped with the digital image acquisition system DISS5 (point electronic GmbH, Halle, Germany).

### Transmission electron microscopy (TEM)

TEM was performed in bovine ampullae revealing severe inflammation (n = 5) as well as in controls (n = 3). 0.5 cm of closed ampulla were fixed in 4% glutaraldehyde in Soerensen’s buffer (1:5 solution of 0.07 M KH_2_PO_4_ and 0.07 M Na_2_HPO_4_.2H_2_O, pH 7.4, 60 mins, RT) and then transferred to 2.5% glutaraldehyde in Soerensen’s buffer (2 hrs, 4 °C). In the following, samples were washed in 0.02 M cacodylate buffer (3 × 5 mins) and in 1% OsO_4_ (Polysciences) and 1.5% K_4_FE(CN)_6_ (4 hrs, RT). After washing in cacodylate buffer and dehydration in an ascending series of ethanols (70%, 80%, 90% and 100%; 3 × 15 mins, RT) specimens were placed in propylene oxide (C_3_H_6_O): epoxy resin at a ratio of 2:1 (60 mins, RT), propylene oxide (C_3_H_6_O): epoxy resin at a ratio of 1:1 (12 hrs, RT), propylene oxide (C_3_H_6_O): epoxy resin at a ratio of 2:1 for (60 mins, RT) and finally in pure epoxy resin. For initial evaluation semithin sections (0.5 µm) were cut and stained with methylene blue. Then ultrathin sections (90 nm) were cut, placed on copper grids and contrasted with a saturated solution of uranyl acetate (UO_2_(CH_3_COO)_2_.2H_2_O) (10 mins, RT). Sections were then washed briefly in dH_2_O, dried and examined using a Zeiss EM 900 microscope at magnifications of x5,000 to x30,000. For documentation and analysis the Slow Scan CCD Camera 7899 (TRS, Moorenweis, Germany) and the software Image SP 1.2.8.111 (Sys Prog, Minsk, Belarus) were used.

### Sample preparation for live cell imaging (LCI)

Qualitative and quantitative analyses in bovine ampullae showing mild, moderate or severe inflammation (n = 45) as well as in healthy controls (n = 10) were performed by digital live cell imaging. 1 cm part of the ampulla of each sample was cut and opened longitudinally. Specimens were pinned onto a Delta T dishes (Bioptechs Inc., PA, USA) coated with Sylgard® (Dow Corning, MI, USA). All samples were maintained at 37.5 °C using a stage heater (Delta T Controller, Bioptech Inc., USA) and an objective lens heater (Delta T Controller, Bioptech Inc., USA) attached to the microscope. Reference temperature was monitored prior to all recordings. For sperm-oviduct interaction and average survival time, 600,000 sperm (30 μl) were coincubated with the ampulla for 10 mins at 37 °C and submerged with sperm Talp before analysis. Imaging was performed with a fixed stage microscope, upright Olympus microscope (BX51WI) with water immersion dipping objectives equipped with the bright-field long-distance immersion objectives UMPLFLN 10xW, UMPLFLN 20xW and UMPLFLN 40xW (Olympus, Hamburg, Germany). Images and videos were documented with a SUMIX Mx7 camera (Summix, CA, USA).

### Quantitative analysis of ciliary beat frequency (CBF)

CBF was assessed in cows revealing mild (n = 15), moderate (n = 15) and severe (n = 15) inflammation in the oviducts as well as in controls (n = 10). CBF was recorded for 5 ROIs with clear motile cilia which were randomly selected. Specimens were imaged with a 40x water immersion objective (UMPLFL 40x W/0.8, Olympus, Hamburg, Germany). The videos were recorded using software from StreamPix® 7.0 (NorPix, Canada) linked to a SUMIX Mx7 camera (100 frames/s (FPS) mounted on a BX51WI fixed-stage upright microscope (Olympus, Hamburg, Germany). Using the Fast Fourier Transformation (FFT) with AutoSignal® (Systat Software, GmbH), CBF was calculated as differences in the shades of grey due to ciliary beat with the use of Image-Pro Plus® software (Media Cybernetics, Inc.).

### Quantitative analysis of particle transport speed (PTS)

Basal PTS was assessed in oviducts with mild (n = 11), moderate (n = 12) and severe (n = 11) inflammation as well as in healthy controls (n = 11). All samples were imaged using a 20x water immersion objective (UMPLFL 20x W/0.5, Olympus, Hamburg, Germany Olympus, Hamburg, Germany) using the StreamPix® 7.0 55 (NorPix, Canada).

For analysis of PTS, 3 μl of dynabeads (approximately 9 × 10^6^ polystyrene beads) with a diameter of 2.8 μm (Life Technologies, AS, Norway) were added to the specimens. To keep the particles from settling within the ampullar folds, the buffer was gently mixed with pipette 3 times and allowed to settle for exactly 1 min prior to every recording. 3 ROIs were selected randomly where 2 oviductal folds were in view and in focus. After conversion of the videos from 12-bit to 8-bit grey scale PTS analysis was performed by automatic tracking using the ImagePro® software (MediaCybernetics, PA, USA).

### Quantitative analysis of sperm motility and average survival time in ampulla

Ampullae revealing mild (n = 8), moderate (n = 8), severe (n = 8) inflammation and controls (n = 7) were coincubated with 600,000 sperm (30 μl) for 10 mins at 37 °C. The formation of the sperm reservoir was recorded using the software StreamPix® 7.0 (NorPix, Canada) linked to a SUMIX Mx7 camera (100 frames per second (FPS)). For each specimen, 5 ROIs with sperm binding to the tube were randomly selected and recorded at time points 0 min, 30 mins, 60 mins, 120 mins and 180 mins. The total number of motile and immotile bound sperm was counted using ImagePro® software (MediaCybernetics, PA, USA).

### Statistical analyses

Statistical analysis was performed by using SPSS 24.0 (IBM, New York. US) and MedCalc 17.9 (Ostend, Belgium). Metric data were tested for normality by the use of D’Agostino Pearson test. All data were normally distributed. All analyses were performed as two-tailed tests. Thickness of tubal folds in healthy and inflamed oviducts was compared using the ANOVA with pairwise multiple comparison tests according to Dunnett. The linear mixed model was used to compare the mean CBF and PTS as well as the mean coefficient of variation of CBF and PTS in healthy and inflamed oviducts. Within the linear mixed model procedure a post hoc test according to Bonferroni was used to compare CBF and PTS between the control and the different grades of inflammation. Further to that the correlation coefficient Spearman’s rho was calculated. The Goodmann and Kruskal’s gamma was used to assess the accumulation of melanin in different grades of inflammation. Additionally the calculation of the area under the curve (AUC) and the two-sided T test for independent samples were used to compare the average number of motile sperm and mean number of sperm bound in the different grades of inflammation and healthy controls.

P < 0.05 was deemed significant and p ≤ 0.001 was considered as highly significant.

## Supplementary information

supplementary information_Movie legends

Effect of inflammation on tubal microarchitecture

Microarchitecture of a healthy control oviduct

Calculation of particle transport speed in a healthy control oviduct

Particle transport speed in an oviduct revealing severe inflammationg

Sperm binding in a healthy oviduct

Sperm binding in an oviduct with severe inflammation
