# Supplementary material for: Salpingitis Impairs Bovine Tubal Function and Sperm-Oviduct Interaction
Source: Sci Rep. 2019 Jul 26;9:10893. doi: 10.1038/s41598-019-47431-x (PMC6659645; doi:10.1038/s41598-019-47431-x)
Supplement: Supplementary file 1 — supplementary information_Movie legends [file 41598_2019_47431_MOESM1_ESM.docx]

Salpingitis Impairs Bovine Tubal Function and Sperm-Oviduct Interaction

Loveth E. Owhor^1^, Sven Reese ^2^ and Sabine Kölle^1*^

^1^School of Medicine, Health Sciences Centre, University College Dublin (UCD), Dublin, Ireland

^2^School of Veterinary Medicine, Institute of Veterinary Anatomy, Histology and Embryology, LMU, Munich, Germany
^*^Correspondence:+35317166636,email:sabine.koelle@ucd.ie itagar.eseoghene@ucdconnect.ie

s.reese@anat.vetmed.uni-muenchen.de

**Supplementary information**

**Movie legends**

Video 1: Oviducts with severe inflammation show areas with loss of tight junctions, demarcation of cells and loss of cilia.

Video 2: In a healthy cow cells are tightly packed and reveal a regular distribution of ciliated cells.

Video 3: Particle transport speed (PTS) is calculated by tracking polystyrene dynabeads in the ampulla of a healthy cow.

Video 4: In an ampulla from a cow with severe inflammation dynabeads are moving slowly. The higher the grade of inflammation the lower is the PTS.

Video 5: In the oviduct of a healthy cow spermatozoa bind to the ciliated cells in a tangential angle with active tail movement.

Video 6: In the oviduct of a cow with severe salpingitis the majority of sperm is immotile and is lying flat on the tubal epithelium.
